# Supplementary material for: Maternal body mass index and the risk of early-onset Group B Streptococcus disease in newborns: A systematic review and meta-analysis
Source: PLoS One. 2026 May 8;21(5):e0329423. doi: 10.1371/journal.pone.0329423 (PMC13155626; doi:10.1371/journal.pone.0329423)
Supplement: S1 Table — (PDF) [file pone.0329423.s001.pdf]

S1 Appendix

Search string from Medline, CENTRAL and Embase Classic+Embase <1947 to 2024 August 5>

	EMBASE	Medline	CENTRAL
1	exp high risk pregnancy/	exp Pregnancy, HighRisk/	MeSH descriptor: [Pregnancy] explode all trees
2	exp pregnancy/	exp Pregnancy/	MeSH descriptor: [Pregnancy Complications] explode all trees
3	exp Pregnancy Complications/	exp Pregnancy Complications/	MeSH descriptor: [Pregnancy, High-Risk] explode all trees
4	exp childbirth/	exp Parturition/	MeSH descriptor: [Parturition] explode all trees
5	"maternal".ti,ab,kf.	"pregnan*".ti,ab,kf.	("pregnancy"):ti,ab,kw (Word variations have been searched)
6	pregnan*.ti,ab,kf.	"maternal".ti,ab,kf.	(maternal):ti,ab,kw (Word variations have been searched)
7	(childbirth or partus or birth or lab?r or obstetric).ti,ab,kf.	(childbirth or partus or birth or lab?r or obstetric).ti,ab,kf.	(childbirth or partus or birth or labor or labour or obstetric):ti,ab,kw
8	1 OR 2 OR 3 OR 4 OR 5 OR 6 OR 7	1 OR 2 OR 3 OR 4 OR 5 OR 6 OR 7	1 OR 2 OR 3 OR 4 OR 5 OR 6 OR 7
9	exp morbid obesity/	exp Body Weight/	MeSH descriptor: [Obesity] explode all trees
10	exp Body Mass Index/	exp Obesity, Morbid/	MeSH descriptor: [Pregnancy in Obesity] explode all trees
11	exp maternal obesity/	exp Obesity, Maternal/	MeSH descriptor: [Obesity, Morbid] explode all trees
12	exp obesity/	exp Obesity/	MeSH descriptor: [Body Mass Index] explode all trees
13	exp body mass/	exp Body Mass Index/	MeSH descriptor: [Ideal Body Weight] explode all trees
14	"obes*".ti,ab,kf.	"BMI".ti,ab,kf.	(overweight):ti,ab,kw (Word variations have been searched)
15	"BMI".ti,ab,kf.	"obes*".ti,ab,kf.	(obesity):ti,ab,kw (Word variations have been searched)
16	"overweight".ti,ab,kf.	"overweight".ti,ab,kf.	(BMI):ti,ab,kw (Word variations have been searched)
17	(adipositas or adiposity or body weight excess or corpulency or obesitas or fat mass or body weight).ti,ab,kf.	(body mass index or body mass or maternal obesity or morbid obesity).ti,ab,kf.	(adipositas or adiposity or body weight excess or corpulency or obesitas or fat mass or body weight):ti,ab,kw (Word variations have been searched)
18	(body mass index or body mass or maternal obesity or morbid obesity).ti,ab,kf.	(adipositas or adiposity or body weight excess or corpulency or obesitas or fat mass or body weight).ti,ab,kf.	(body mass index or body mass or maternal obesity or morbid obesity):ti,ab,kw (Word variations have been searched)
19	9 OR 10 OR 11 OR 12 OR 13 OR 14 OR 15 OR 16 OR 17 OR 18	9 OR 10 OR 11 OR 12 OR 13 OR 14 OR 15 OR 16 OR 17 OR 18	9 OR 10 OR 11 OR 12 OR 13 OR 14 OR 15 OR 16 OR 17 OR 18
20	exp Streptococcal infections/	exp Streptococcal Infections/	MeSH descriptor: [Streptococcus agalactiae] explode all trees
21	exp Streptococcus agalactiae/	exp Streptococcus agalactiae/	MeSH descriptor: [Streptococcal Infections] explode all trees

22	(group b streptococc* or beta h* streptococcus group b or ha?molytic streptococcus b or hemolytic streptococcus b or staphylococc* agalactiae or streptococcus group B or streptococcus group b or streptococc*	(group b streptococc* or beta h* streptococcus group b or ha?molytic streptococcus b or hemolytic streptococcus b or staphylococc* agalactiae or	(group b streptococcus OR beta h streptococcus group b OR haemolytic streptococcus b OR staphylococcus agalactiae):ti,ab,kw (Word variations have been searched)
	agalactia* or streptococc* infection* or streptococc* coloni?ation* or GBS coloni?ation* or GBS infection* or (GBS adj3 screening) or (streptococc* adj3 b) or streptococc*).ti,ab,kf.	streptococcus group B or streptococcus group b or streptococc* agalactia* or streptococc* infection* or streptococc* coloni?ation* or GBS coloni?ation* or GBS infection* or (GBS adj3 screening) or (streptococc* adj3 b) or streptococc*).ti,ab,kf.	
23	("Early Onset GBS" or EOGBS).ti,ab,kf.	("Early Onset GBS" or EOGBS).ti,ab,kf.	(Early Onset GBS OR EOGBS):ti,ab,kw (Word variations have been searched)
24	20 OR 21 OR 22 OR 23	20 OR 21 OR 22 OR 23	(group b streptococc* or beta h* streptococcus group b or haemolytic streptococcus b or hemolytic streptococcus b or staphylococc* agalactiae or streptococcus group B or streptococcus group b or streptococc* agalactia* or streptococc* infection* or streptococc* colonisation* or GBS colonisation* or GBS infection* or (GBS adj3 screening) or (streptococc* adj3 b) or streptococc*):ti,ab,kw (Word variations have been searched)
25	8 AND 19 AND 24	8 AND 19 AND 24	20 OR 21 OR 22 OR 23 OR 24
			8 AND 19 AND 25
