# Supplementary material for: Maternal body mass index and the risk of early-onset Group B Streptococcus disease in newborns: A systematic review and meta-analysis
Source: PLoS One. 2026 May 8;21(5):e0329423. doi: 10.1371/journal.pone.0329423 (PMC13155626; doi:10.1371/journal.pone.0329423)
Supplement: S2 Table — (DOCX) [file pone.0329423.s002.docx]

**S3 Table.** Predicted risk differences pr 1000 births with 95% confidence intervals based on the main meta regression analysis for additional BMI units above normal level. Reference level for normal weight was 0.43 pr 1000 according to Edmond et al. (2012). With lower and higher baseline risk levels for sensitivity evaluation, supplemented with results for the model estimated on studies reporting adjusted ORs.

| **Baseline risk**  **pr 1000 births**  **BMI 22.3** | **BMI 30** | **BMI 35** | **BMI 40** | **BMI 45** |
| --- | --- | --- | --- | --- |
| 0.32 (75%) | 0.06 (0.02-0.10) | 0.11 (0.04-0.18) | 0.17 (0.05-0.28) | 0.23 (0.06-0.39) |
| 0.43 (Reference) | 0.08 (0.03-0.14) | 0.15 (0.05-0.25) | 0.22 (0.07-0.37) | 0.30 (0.08-0.52) |
| 0.65 (150%) | 0.13 (0.05-0.21) | 0.22 (0.08-0.37) | 0.33 (0.10-0.56) | 0.45 (0.12-0.78) |
| 0.86 (200%) | 0.17 (0.06-0.27) | 0.30 (0.10-0.49) | 0.44 (0.14-0.75) | 0.60 (0.16-1.04) |
| Adjusted OR*  0.43 (Reference) | 0.11 (0.06-0.16) | 0.19 (0.10-0.29) | 0.29 (0.14-0.44) | 0.40 (0.18-0.63) |
