# Supplementary material for: Maternal body mass index and the risk of early-onset Group B Streptococcus disease in newborns: A systematic review and meta-analysis
Source: PLoS One. 2026 May 8;21(5):e0329423. doi: 10.1371/journal.pone.0329423 (PMC13155626; doi:10.1371/journal.pone.0329423)
Supplement: S1 Fig — (DOCX) [file pone.0329423.s005.docx]

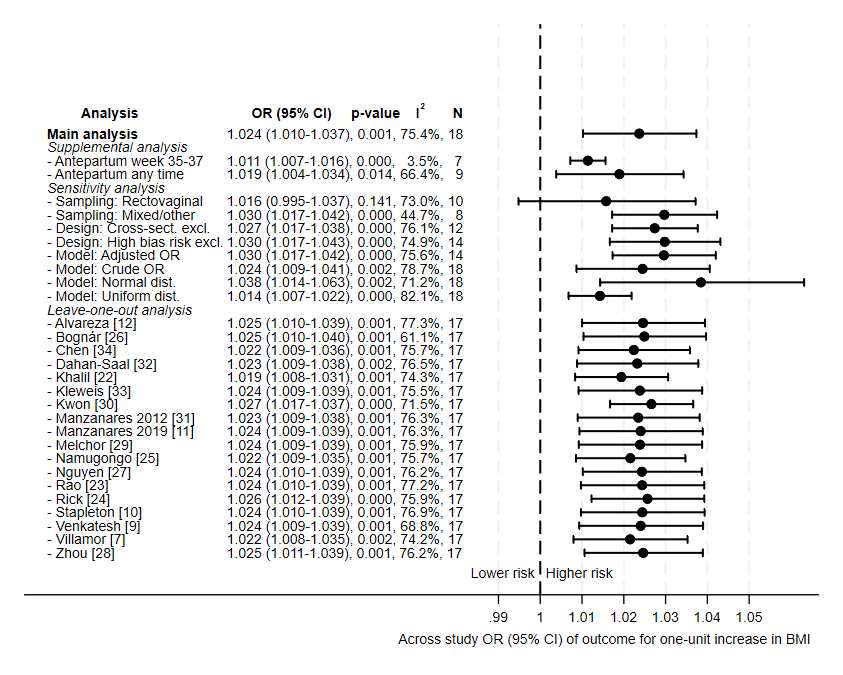


**S4 Figure. Forest plot representing the association between BMI and risk of EOGBS or proxy outcomes by odds ratios with 95% confidence intervals based on random-effects meta-regression. Results representing: Main analysis, supplementary, sensitivity, and leave-one-out analyses.**
