# Supplementary material for: Maternal body mass index and the risk of early-onset Group B Streptococcus disease in newborns: A systematic review and meta-analysis
Source: PLoS One. 2026 May 8;21(5):e0329423. doi: 10.1371/journal.pone.0329423 (PMC13155626; doi:10.1371/journal.pone.0329423)
Supplement: Attachment — Submitted filename: Response to reviewers.docx [file pone.0329423.s007.docx]

# Response to the Editor and Reviewers

Manuscript title:

Maternal Body Mass Index and the Risk of Early-Onset Group B Streptococcus Disease in Newborns: A Systematic Review and Meta-Analysis

Journal: PLOS ONE

Authors: Elisabeth Emanuel Graae et al.

## Rebuttal Letter

We sincerely thank the Editor for the thoughtful and constructive feedback. We have revised the manuscript accordingly and believe that these changes have substantially improved the clarity, transparency, and methodological rigour of our work. Below, we reproduce each comment in bold followed by our response in italics. Page and line numbers refer to the clean version of the revised manuscript.

### Editorial and Journal Requirements

- **Formatting according to PLOS ONE style**

Response: *The entire manuscript has been revised to follow PLOS ONE’s Word-template style. Heading levels, table captions, and figure captions have been standardized.*

- **Data Availability Statement**

Response: *We have updated the Data Availability Statement to confirm that all raw data underlying the analyses (study-ident, BMI categories, 95% CIs) are available as Supporting Information S5 File (CSV) data set.*

- **LaTeX template**

Response: *It appears from the online manuscript instructions that PLOS ONE accepts Word submissions, we have thus retained the Word format but ensured that all structural and reference-style elements comply with PLOS guidelines. It LaTeX is required please let us know and we will update accordingly.*

- **Figure captions**

Response: *Each figure (Figs 1–5) now includes a complete, self-contained legend in the file title.*

- **Citation of recommended references**

Response: *No additional references has been suggested.*

### Scientific and Methodological Comments

- **BMI categorization and transformation**

Response: *A new Sensitivity Analysis subsection has been added (Methods 2.5 and Results 3.1). We evaluated alternative BMI-distribution assumptions (normal, uniform). The effect estimates remained stable (Fig 3), confirming robustness.*

- **Inclusion of different study designs**

Response: *We now explicitly discuss the implications of combining cohort, cross-sectional, and case-control designs (Discussion 4.1, p. 17-18). A sub-analysis comparing study designs (Fig 3) produced similar pooled estimates, suggesting that heterogeneity is not driven by design type.*

- **Outcome definitions and testing methods**

Response: *Sections 2.5 and 3.1 have been expanded to describe variability in GBS testing (culture vs PCR; rectovaginal vs urine). Subgroup results are illustrated in Fig 3. No major directional differences were found.*

- **Adjustments and confounders**

Response: *We now specify that fully adjusted models were prioritized in the main analysis. We had no pre-specified approach (Methods 2.5, p. 6). This rationale and resolvement is discussed in Discussion 4.1 (p. 17-18).*

- **Statistical diagnostics**

Response: *The Methods 2.5 section now notes that funnel and residual plots were inspected for symmetry and homoscedasticity (see Fig 5). No violations of model assumptions were observed.*

### Closing Statement

We hope that the revisions fully address the editorial concerns. We are grateful for the opportunity to revise and resubmit this work.

Kind regards,
Elisabeth Emanuel Graae, MD (Corresponding Author)
on behalf of all co-authors
Department of Gynecology and Obstetrics, Lillebaelt University Hospital
elisabeth.emanuel.graae@rsyd.dk

Cover Letter for Editorial Manager

Dear Editor,

Please find enclosed our revised manuscript entitled “Maternal Body Mass Index and the Risk of Early-Onset Group B Streptococcus Disease in Newborns: A Systematic Review and Meta-Analysis.” We have carefully addressed all comments, as detailed in the attached Rebuttal Letter. The revised version includes expanded methodological descriptions, sensitivity analyses, and updated data-sharing information in full compliance with PLOS ONE requirements.

We sincerely thank you for considering our revision and look forward to your evaluation.

Best regards,

Elisabeth Emanuel Graae, MD
Corresponding Author
