# Supplementary material for: Maternal body mass index and the risk of early-onset Group B Streptococcus disease in newborns: A systematic review and meta-analysis
Source: PLoS One. 2026 May 8;21(5):e0329423. doi: 10.1371/journal.pone.0329423 (PMC13155626; doi:10.1371/journal.pone.0329423)
Supplement: Attachment — Submitted filename: 260312NU Response to Reviewers_2.docx [file pone.0329423.s008.docx]

# Response to the Editor and Reviewers

Manuscript title:

Maternal Body Mass Index and the Risk of Early-Onset Group B Streptococcus Disease in Newborns: A Systematic Review and Meta-Analysis

Journal: PLOS ONE

Authors: Elisabeth Emanuel Graae et al.

Thank you very much for your thoughtful and constructive feedback. We have carefully addressed all comments, as detailed in the point-by-point responses below. In doing so, substantial portions of the manuscript were revised, which we believe has significantly improved its clarity and readability.

In response to the reviewers’ suggestions, we conducted an updated search that led to the inclusion of three additional studies. We have also added a new table (Table 4) to provide a clearer overview and included a supplementary table reporting absolute estimates (Table S3) to better contextualize our results.

Our detailed responses are provided below, with our replies indicated in italics.

**Reviewer #1:** The subject addressed in this systematic review is of considerable importance. Unfortunately, there are major weaknesses in the report. The authors may find the Handbook produced by the Cochrane Collaboration (cochrane.org/authors/handbooks-and-manuals/handbook/current) helpful. My detailed comments are as follows:

MAJOR

1. p.3, ll.2-4. For the benefit of non-clinical readers, more background information should be given regarding Group B Streptococcus and, specifically, EOGBS. In addition, the distinction between parity and gravidity should be described.

Response: *Thank you for this helpful suggestion to improve readability for non-clinical readers. We have expanded the Background section to provide additional context on* Group B Streptococcus *and early-onset Group B streptococcal disease (EOGBS), with particular emphasis on the hypothesized mechanisms linking maternal obesity and EOGBS.*

*In addition, we have clarified the meaning of parity by adding “(number of prior births)” at its first occurrence in the text. The term* gravidity *is not used in the manuscript.*

2. p.4, ll.19-20. The time periods covered by the MEDLINE, Embase and CENTRAL databases should be stated. Given the date of the search (5th August 2024), it needs to be re-run.

Response: *An updated search was run on the 28^th^ of January 2026, and the time period covered was stated. 3 new studies were retrieved and incorporated in the review and meta-analysis.*

3. p.6. References should be given for random-effects meta-regression (l.3) leave-one-out analysis (l.19) and the I-squared statistic (bottom line).

Response: *We appreciate this comment. These references have been provided in the Statistical Analysis section, 2.5.*

4. pp.9-10, Table 1. The reference numbers given in the first column do not match the corresponding numbers in Tables 2 and 3, e.g. Rao (2019) is [13] in Table 1 but [14] in Tables 2 and 3. In Column 4, "N (cases)" might be clearer as "Sample size (cases)".

Response: *See response 6*

5. pp.11-13, Table 2. All papers cited in this table should be carefully checked for content as important information is missing, e.g., in Rao (2019) the percentages for the ethnic groups considered are reported (White British 27.4%, White other 32.0%, etc). NB I have not checked all the papers. At the top of Column 6, what does "P0 %" mean? The information on socio-economic status (Column 8) is confusing, as education, marital status, an index of deprivation and type of insurance are all in this single column. The demographic information should be presented in an additional table, with separate columns for education, marital status, etc. For Rao (2019), the acronym IMD should be explained.

Response: *See response 6*

6. pp.13-15, Table 3. The information presented in this table is very dense, and the Main Results (Column 4) are particularly difficult to identify. aHR, aOR and OR should be explained. There should be separate columns for BMI category, the estimate, and 95% confidence interval. This might be easier to achieve with the table in landscape format.

Response (4, 5 and 6): *We thank the reviewer for drawing attention to the formatting errors in Table 1 which are now corrected and aligned with the reference list.* *Column 4 in Table 1 has been renamed following the advice from #1 reviewer. Table 2 and Table 3 have been rearranged for easier identification of the main outcome. Extracted information in Table 2 was reassessed with a few modifications and additions. Table 3 has been reformatted in landscape orientation to enhance readability and reduce visual density. In line with the reviewer’s suggestion, we have separated the BMI category, effect estimate, and 95% confidence interval into distinct columns to improve transparency and readability. This restructuring makes the reported associations clearer and allows for easier comparison across studies. The adjusted covariates in the multivariate analysis were removed from Table 2 to Table 3 to emphasize the potential confounders considered. Regarding Table 2, P0 % was explained and the specific percentages of the population in Rao (2019) were applied. Regarding socio-economic status (Column 8), we acknowledge that presenting education, marital status, deprivation indices, and insurance type within a single column may appear condensed. We carefully considered restructuring this information into separate columns, as suggested. However, due to substantial heterogeneity in how socio-economic variables were defined and reported across studies—and in many cases very limited or incomplete reporting—creating separate columns would have resulted in a sparse and fragmented table with substantial missing data. To balance completeness and readability, we have retained a single socio-economic column but revised its information with explanation of IMD in the table footnote to improve clarity.*

*We are grateful for these constructive suggestions, which have significantly improved the clarity, consistency, and presentation of the tables in the manuscript.*

7. p.19, ll.20-21. I find this statement regarding the preference for a crude OR concerning, given that there is potentially a high risk of seriously misleading findings if the effects of other appropriate explanatory variables are ignored.

Response: *We agree that this statement is misleading. It has been deleted in the revised manuscript.*

8. p.21, ll.4-6. A clear distinction should be drawn between statistical significance and clinical importance. In determining the clinical importance of a finding, the limit of the 95% confidence interval closer to the null hypothesis value, in this case the lower limit, should be considered.

Response: *Thank you for this important comment. We certainly agree that the confidence interval should be considered when evaluating the clinical significance of a statistically significant finding. This is now emphasized tin the conclusion: “*This systematic review and meta-analysis suggests a modest but consistent association between increasing maternal BMI and the risk of GBS colonization and early-onset GBS disease. Therefore, the integration of BMI into existing risk assessment models warrants a formal health technology assessment.”

*Further, we have expanded the presentation of the results. Specifically, we added* ***Table 4****, which reports estimated ORs across selected BMI categories based on the pooled estimate from the meta-analysis. In addition, we added* ***Supplementary Table S3****, which presents the corresponding* ***absolute risk differences*** *for these BMI levels. Presenting the results in absolute terms allows the clinical magnitude of the association to be more easily interpreted.*

*These additions were intended to contextualize the findings and facilitate assessment of their potential clinical importance. The relevant description and interpretation have been added to the Results section (Page 19-20, lines 269–279). Moreover, this focus is applied in the discussion (Page 22-23, Lines 328-336).*

9. In Figure 1 (PRISMA flow-chart), the numbers of records identified, removed because of duplication and screened are incompatible, i.e., 844 + 491 + 311 + 42 - 624 is not equal to 689. Is there a typing error here?

Response: *We thank the reviewer for helping us discovering this inconsistency. The PRISMA diagram has been updated accordingly.*

MINOR

1. p.5, l.12. The role of the individuals involved in the third-party adjudication should be stated, e.g., were they part of the project team? Did they input into other aspects of this work?

Response: *Thank you for helping us strengthening the transparency of out methodology. This has now been described accordingly in the Methods section.*

2. p.5, l.12. A reference should be given for the Covidence software.

Response: *Thank you for pointing this out. A reference for the Covidence software has been added.*

3. p.5, ll.16-17. The primary outcome and each proxy outcome should be listed here using bullet points.

Response: *Thank you for this suggestion. The Methods section has been reorganized, and the primary and proxy outcomes now stand out more clearly in section 2.2 Eligibility criteria.*

4. p.5. Were the corresponding authors of the relevant reports contacted in order to obtain additional information?

Response: *Thank you for this comment. Data extraction was based on the information reported in the articles and their supplementary materials. In one case where additional details were of crucial interest, the corresponding authors were contacted (Kleweis et al.); however, no response was received. Therefore, the analysis relied on the data available in the published sources which has been specified in the 2.4 Study Selection and Data Extraction section.*

5. p.29. Should "syntesis" be "synthesis"?

Response: *Yes. Thank you for highlighting this error which has been corrected.*

6. The Figures are not presented in the correct order.

Response: *It was carefully checked that figures are presented in the correct order in the revised version of the manuscript.*

7. In Figure 4 (QUIPS study quality assessment) what do the green and light orange circles represent?

Response: *Thank you for helping us clarifying this. Explanatory definitions have now been added to the QUIPS study quality assessment (Fig. 4).*

**Reviewer #2:** The manuscript addresses a pertinent clinical question and is generally well organized. However, I have four comments regarding reporting that could enhance clarity and methodological transparency.

1. Citation Numbering in Table 1
In Table 1, the numeric citation labels for several studies do not match the reference list numbering. For instance, Namugongo 2016 is labeled as “(15)” in Table 1, while it is referenced as “(16)” in the reference list. In contrast, the citations in the other tables are accurately numbered, which suggests a formatting issue specific to Table 1. To prevent misunderstandings for readers who wish to verify the studies included, please correct Table 1 to ensure that the citation numbering aligns with the reference list.

Response: *We thank the reviewer for noticing this formatting error in Table 1 that has been corrected and aligned with the reference list.*

2. PRISMA Flow Diagram Arithmetic
The PRISMA flow diagram shows an inconsistency in the numbers reported for identified records, duplicates removed, and records screened. Specifically, the diagram indicates Records identified (n = 844), Duplicates removed (n = 624), and Records screened (n = 689); however, (844 − 624 does not equal 689) . This step needs to be recalculated, and the PRISMA diagram should be updated accordingly.

Response: *We thank the reviewer for helping us discovering this inconsistency. The PRISMA diagram has been updated accordingly.*

3. Clarification of BMI Measurement Timing
It would be beneficial to clarify whether BMI was assessed prior to pregnancy, at first antenatal booking, or at later stages of gestation for each of the included studies. These time points are not interchangeable clinically, and pooling these measures without clear delineation may lead to exposure misclassification. Even if subgroup analysis is not feasible, specifying the timing in the Methods or Tables and briefly discussing this limitation in the Discussion section would enhance interpretability.

Response: *This is an important notice. As defined in our eligibility criteria the prognostic factor is pregestational BMI (assessed within 10^th^ week of gestation). For most studies it remains unclear if BMI was measured at first antenatal visit by a health professional or reported by the patient. In the “comments” column in Fig 2, this issue is further described. We considered this concern and chose to downgrade the quality of evidence on “Prognostic factor” in the studies lacking information on timing of BMI assessment. More information on this is available in 2.6 “Quality assessment” which was rewritten to improve transparency and methodology. Moreover, the issue was briefly addressed in section 4.3.*

4. Funnel Plot Description and Interpretation
In describing Fig. 5, it states that it represents a funnel plot of individual study log odds ratios against their standard errors to assess publication bias. However, the axes appear to depict residuals versus standard error, which aligns more closely with influence diagnostics than with a traditional funnel plot. Additionally, the figure label claims the plot is based on “all included studies,” yet it remains unclear which outcome(s) these studies pertain to. For the primary outcome (EOGBS), the small number of included studies (approximately k = 5) typically does not provide a robust basis for funnel plot analysis. Clarifying the dataset represented and tempering the interpretation would significantly enhance the reporting.

Response: *We thank the reviewer for drawing attention to this. The description is misleading in this context with a regression based funnel plot. We have corrected the text and figure captions in Fig. 5 accordingly.*

**Reviewer #3:** Methods
• Section 2.3: the statement that no studies were yielded from the grey literature/citation tracking should not be found in the methods. I suggest omitting or moving to the results. There is an extra period after “AND.” Inconsistent capitalization of medical subject headings. Suggest defining MeSH as it is first introduced without defining the acronym. Additional information on grey literature search would be appreciated (e.g., how many studies searched, what was searched).

Response: *Thank you for these constructive suggestions. The statement that no studies were yielded from the grey literature/citation tracking has been moved to the results section. Additional information on grey literature search was provided in subsection 2.3. The MeSH acronym has been defined at first appearance in the manuscript.*

• Section 2.4: The entire section on PFO criteria should be moved to the eligibility criteria. The criteria should be presented in a paragraph, not in bulleted text. Clarify who was responsible for “third-party adjudication” and if data extraction was performed in duplicate (if so, if there was a process for resolving discrepancies for data extraction).

Response: *Thank you for helping us clarifying the methodology and improving transparency. The eligibility criteria were properly defined in section 2.2, and the processes including specification of the “third-party adjudication” were clarified. We hope that the 2.2 section now stands out clear since further description of the inclusion criteria in the paragraph was added. However, the PFO criteria were kept in bulleted text for an easy overview.*

• Section 2.5: citation for the source of the numbers log 24.6 and log 1.2. OR needs to be explicitly defined. If there were prespecified thresholds for heterogeneity, they should be specified. What does “No major violations were identified” refer to? It’s not clear and also sounds like a result, not a prespecified method. Specify packages used in Stata for the analysis. Unclear what the purpose of a sensitivity analysis of OR < 2 is for.

Response: *Thank you for these helpful comments. The manuscript has been revised accordingly. A citation for the source of the values log(24.6) and log(1.2) has now been added in Section 2.5. The term odds ratio (OR) is now explicitly defined at its first appearance in the text. We agree that the sentence “No major violations were identified” was not appropriate for the Methods section, and it has therefore been removed. In addition, the Stata packages used for the analyses are now specified in Section 2.5. Finally, we have clarified the purpose of the sensitivity analysis restricted to OR < 2 in section 2.5.*

• Strongly recommend adopting the GRADE framework for assessing certainty in the evidence for improved interpretation of the findings. It would also be beneficial to have absolute risk differences to help contextualize the absolute magnitude of some of the relative effect estimates you find.

Response: *We appreciate the reviewer’s strong recommendation to adopt the GRADE framework to assess certainty in the evidence. While GRADE is highly valuable—particularly for informing clinical recommendations—our primary aim in this review was to evaluate and synthesize the association between maternal obesity and GBS-related disease risk rather than to issue clinical practice guidance.*

*Nonetheless, we agree that an assessment of certainty in the evidence would enhance interpretability. At this stage, a full GRADE assessment was not implemented. However, we have more explicitly addressed the overall certainty and limitations of the evidence base, including study design, residual confounding, and imprecision in section 4.3.*

*In addition, we agree that presenting absolute risk differences improves clinical interpretability. We have added absolute risk difference in the Results section with a new supplementary table (S3 Table). Specifically, we now report that the risk of EOGBS was 0.86 per 1000 live births among individuals with BMI 40 compared with 0.43 per 1000 among those with normal BMI (22.3), corresponding to an absolute risk difference of approximately 0.22 per 1000 births. The text has been revised accordingly (page 20, lines 283-286).*

• Section 3.1: Avoid using ‘e.g.’ in a sentence. For the categorization of GBS assessment timing, specify the number and % of studies for each. In the sentence describing geographical region, use textual numbers for all for consistency and because the values < 10. P values should be reported throughout. I2 should be provided for major results in the text. What does “exponential increase” in OR mean? The main finding (OR 1.027) needs to be clearly reported in the text with 95% CI. All subgroup analyses should report a p value for interaction to quantify whether there are significant differences between subgroups.

Response: *The number and percentage for timing of GBS assessment was specified in section 3.1. Furthermore, the studies are presented in their respective categories in the result tables for an easy overview. All reasons for study exclusion were named (instead of giving examples). The reviewer’s suggestion for reporting p values in the subgroup analyses is appreciated. P values are now reported in the subgroup analyses in Fig. 3 to enhance comparability as well as for the main results. Main findings are presented with 95% CI throughout, and I2 for most important findings were provided in the Results section. Moreover, textual numbers were applied for consistency as suggested. The expression “expontential increase” was based on a visual interpretation, and we agree that this is misleading and inappropriate. For this reason, it has been removed. Moreover, the following sentence has been added in describing our main findings: “*Assuming a log-linear association, each one-unit increase in BMI was associated with 2.4% higher odds of EOGBS and proxy outcomes (OR 1.024, (95% CI 1.010-1.037; p=0.001” (*page 19, lines 259-261).*

• Section 3.2: details about RoB assessment should be first introduced in the methods. A citation is needed for the QUIPS tool. Specify the authors who conducted the assessment and what process was used to resolve conflicts. Define what your overall risk of bias levels are for the studies and how these were determined from the individual domains. Specify the number and % of the low RoB studies when you refer to “most studies.” I have serious doubts about the quality of the QUIPS assessment. All studies are rated to be at a low risk of bias due to confounding, but many studies report unadjusted estimates which are very vulnerable to confounding.

Response: *We thank the reviewer for these important methodological comments. To address these concerns, we have made several revisions to the manuscript.*

*First, a new subsection (Section 2.6) has been added to the Methods section, where the risk of bias (RoB) assessment is now introduced prior to the presentation of results. In this subsection, we describe the use of the QUIPS (Quality In Prognosis Studies) tool and provide the appropriate citation. The QUIPS tool was selected following consultation with Cochrane due to its suitability for prognostic factor research and observational study designs, which align with the objective of our review.*

*Second, we have clarified that two independent reviewers conducted the RoB assessments. Discrepancies were resolved through discussion until consensus was reached. This process is now explicitly described in the Methods section.*

*Third, we have defined how overall risk of bias judgments were derived from the six QUIPS domains. We have also specified the exact number and proportion of studies classified as low risk of bias etc.*

*Regarding the reviewer’s concern that all studies were rated as low risk of bias due to confounding despite reporting unadjusted estimates: We carefully re-evaluated this domain. In fact, two studies (Rao 2019 and Kwon 2023) from the original manuscript reported only unadjusted estimates. These studies have now been reassessed and downgraded in the confounding domain and, consequently, in the overall risk of bias rating. Yet two more studies reporting only unadjusted estimates were identified in the updated search. For these, the same grading strategy was used. We agree that unadjusted analyses are inherently vulnerable to confounding and have clarified in the manuscript how confounding was evaluated within QUIPS. We believe these revisions strengthen the transparency and credibility of the RoB assessment.*

• Figure 1: the numbers in records identified, duplicates removed, and records screened to not add up.

Response: *We thank the reviewer for helping us discovering this inconsistency. The PRISMA diagram has been updated accordingly.*

• Figure 3: suggest avoiding arrows at the end of 95% CIs unless the CI extends beyond the axis labels. Also suggest having a separate forest plot for the pooled analysis that shows the ORs of the individual studies along with the pooled estimate. This new forest plot should show the weights of the included studies.

Response: *Figure 3 has been replaced by a new plot which include the individual studies with their random effects coefficients from the meta regression analysis, as such representing the level and study uncertainty. Direct Information on weights is not available as in a typical meta-analysis as each study is represented by the study reported BMI category ORs with confidence intervals. Furthermore, arrows have been replaced by vertical lines.*

Discussion
• Section 4.3: further discussion on heterogeneity is warranted. For example, the study characteristics show notable difference in racial composition, and GBS colonization rates and BMI may vary across these populations.

Response: *We thank the reviewer for these important comments. The discussion has been revised to more explicitly address sources of clinical and methodological heterogeneity, including study design, GBS assessment, BMI measurement, and variation in confounder adjustment. We have also expanded the discussion of risk of bias, particularly residual confounding, misclassification, and limitations related to causal inference (Page 24-25, section 4.3).*

• An exploration of physiological reasons why EOGBS may be increased in patients who are obese would be relevant.

Response: *We agree. This has been more explicit addressed in the Background (page 3-4, lines 70-81), and remains available in the Discussion (page 23, lines 337-344). Finally, it has been emphasized in section 4.3 that our findings demonstrate association rather than causation (page 25, lines 383-385).*

Supplemental information
• Provide the number of results per line in all search strategies

Response: *Thank you for this comment. We understand that you would like us to report the number of results retrieved for each individual search line of each database.*

*However, bibliographic databases are dynamic, and the number of hits retrieved for individual lines may change from day to day, even when the search strategy remains unchanged. Reporting line-by-line results at this stage would not necessarily match the numbers presented in the PRISMA flow diagram, which reflects the total number of records identified per database at the time the final searches were conducted.*

*Our reporting follows the recommendations of PRISMA-S (Preferred Reporting Items for Systematic Reviews and Meta-Analyses – Search extension), which emphasizes transparent reporting of the full search strategy for each database, including exact search strings and limits used. PRISMA-S does not require reporting the number of results for each individual search line; instead, it reports the total number of records retrieved per database, which we have provided in the PRISMA flow diagram.*

*In accordance with established methodological standards and Cochrane guidance, systematic reviews do not report the number of records retrieved for each individual search line. Even Cochrane reviews—the recognized gold standard—report only the full reproducible search strategies and the total number of records per database, not per search line.*

*Based on this, and consistent with PRISMA-S recommendations, we respectfully disagree that line-by-line counts should be provided. The current reporting accurately reflects total database yields while maintaining transparency, reproducibility, and adherence to internationally accepted standards for systematic reviews.*

• Ensure that Table S5 is CSV, it appears to be a Numbers file
• Line numbers, which are required by PLOS One, would be very helpful for review comments
• Suggest adding a space between < or > and the following number throughout

Response: *Thank you for these three helpful suggestions which have all been applied.*

*We thank the reviewers for these constructive suggestions, which have substantially strengthened the methodological transparency and interpretability of our review.*
