# Supplementary material for: Maternal body mass index and the risk of early-onset Group B Streptococcus disease in newborns: A systematic review and meta-analysis
Source: PLoS One. 2026 May 8;21(5):e0329423. doi: 10.1371/journal.pone.0329423 (PMC13155626; doi:10.1371/journal.pone.0329423)
Supplement: Attachment — Submitted filename: Response to reviewers_3.docx [file pone.0329423.s009.docx]

# Response to the Editor and Reviewers

Manuscript title:

Maternal Body Mass Index and the Risk of Early-Onset Group B Streptococcus Disease in Newborns: A Systematic Review and Meta-Analysis

Journal: PLOS ONE

Authors: Elisabeth Emanuel Graae et al.

We thank the reviewers once again for their time; their thorough and valuable feedback is greatly appreciated.

**Reviewer #1**: The manuscript is much better. I have a few minor comments:

1. ll. 199-203. This sentence is difficult to follow. Are the semicolons and commas in the correct positions? A semicolon might be more appropriate after "[22]" and [12,23-28]".

Response: *Thank you for this helpful comment. We agree that the sentence structure was unclear. We have revised the sentence to improve readability and ensure consistent use of punctuation. Specifically, we now use a colon to introduce the categories and semicolons to clearly separate them.*

2. Table 2. There are two instances of "marietal status".

Response: *Thank you for noticing this. These two instances have been corrected in the revised manuscript.*

3. l. 403. The word "diversity" could be interpreted in the context of EDI (Equality, Diversity, and Inclusion). Might "variation between observations" be better?

Response: *Thank you for this suggestion which has been applied in line 403.*

4. ll. 426-427. Is this sentence complete?

Response: *To improve readability this sentence was rewritten.*

**Reviewer #3**: Most of the previous comments appear to be addressed. I still find the pre-planned sensitivity analysis restricting studies based on OR < 2 to be unjustified. Excluding studies solely based on the magnitude of their effect estimates introduces bias; if studies with more extreme effect estimates were thought to be at a higher risk of bias, this would be accounted for in the analysis by study risk of bias. Leave-one-out analyses are already performed to assess the stability of the pooled results. Additionally, there is no justification why a threshold of OR = 2.0 was selected. Your protocol does not state this threshold was established a priori: “Additionally, supplementary sensitivity analyses will be conducted, to evaluate the influence of factors such as large studies on the overall effect measure in cases of heterogeneity.” I would argue that none of the sensitivity analyses that were performed were explicitly pre-planned, and describing them as such may be misleading.
Formatting: line 158 I^2 should be in superscript.

Response: *Thank you for this constructive comment. As the sensitivity analyses were not pre-specified in the protocol, we agree that describing them as pre-planned is inappropriate. This statement has therefore been removed in Section 2.5. Moreover, we acknowledge the reviewer’s concern regarding the restriction of studies based on the magnitude of the effect estimate (OR < 2), and have removed this analysis from both the text and figures.*
*Formatting: Two instances of “I2” were corrected in lines 157 and 158.*
